# Insulin Secretory Defect and Insulin Resistance in Isolated Impaired Fasting Glucose and Isolated Impaired Glucose Tolerance

**DOI:** 10.1155/2016/1298601

**Published:** 2015-12-15

**Authors:** Sae Aoyama-Sasabe, Mitsuo Fukushima, Xin Xin, Ataru Taniguchi, Yoshikatsu Nakai, Rie Mitsui, Yoshitaka Takahashi, Hideaki Tsuji, Daisuke Yabe, Koichiro Yasuda, Takeshi Kurose, Nobuya Inagaki, Yutaka Seino

**Affiliations:** ^1^Division of Clinical Nutrition and Internal Medicine, Okayama Prefectural University, Okayama 719-1197, Japan; ^2^Preemptive Medicine and Lifestyle-Related Disease Research Center, Kyoto University Hospital, Kyoto 606-8507, Japan; ^3^Faculty of Computer Science and Systems Engineering, Okayama Prefectural University, Okayama 719-1197, Japan; ^4^Division of Diabetes and Endocrinology, Kyoto Preventive Medical Center, Kyoto 604-8491, Japan; ^5^Kyoto Institute of Health Science, Kyoto 604-0845, Japan; ^6^Center for Preventive Medicine, St. Luke's International Hospital, Tokyo 104-6591, Japan; ^7^Faculty of Health and Welfare Science, Okayama Prefectural University, Okayama 719-1197, Japan; ^8^Center for Diabetes, Endocrinology and Metabolism, Kansai Electric Power Hospital, Osaka 553-0003, Japan; ^9^Yutaka Seino Distinguished Center for Diabetes Research, Kansai Electric Power Medical Research Institute, Kobe 650-0047, Japan; ^10^Department of Diabetes and Endocrinology, Saiseikai Noe Hospital, Osaka 536-0001, Japan; ^11^Department of Diabetes and Clinical Nutrition, Graduate School of Medicine, Kyoto University, Kyoto 606-8507, Japan

## Abstract

*Objective.* To investigate the characteristics of isolated impaired glucose tolerance (IGT) and isolated impaired fasting glucose (IFG), we analyzed the factors responsible for elevation of 2-hour postchallenge plasma glucose (2 h PG) and fasting plasma glucose (FPG) levels. *Methods.* We investigated the relationship between 2 h PG and FPG levels who underwent 75 g OGTT in 5620 Japanese subjects at initial examination for medical check-up. We compared clinical characteristics between isolated IGT and isolated IFG and analyzed the relationships of 2 h PG and FPG with clinical characteristics, the indices of insulin secretory capacity, and insulin sensitivity. *Results.* In a comparison between isolated IGT and isolated IFG, insulinogenic index was lower in isolated IGT than that of isolated IFG (0.43 ± 0.34 versus 0.50 ± 0.47, resp.; *p* < 0.01). ISI composite was lower in isolated IFG than that of isolated IGT (6.87 ± 3.38 versus 7.98 ± 4.03, resp.; *p* < 0.0001). In isolated IGT group, insulinogenic index showed a significant correlation with 2 h PG (*r* = −0.245, *p* < 0.0001) and had the strongest correlation with 2 h PG (*β* = −0.290). In isolated IFG group, ISI composite showed a significant correlation with FPG (*r* = −0.162, *p* < 0.0001) and had the strongest correlation with FPG (*β* = −0.214). *Conclusions.* We have elucidated that decreased early-phase insulin secretion is the most important factor responsible for elevation of 2 h PG levels in isolated IGT subjects, and decreased insulin sensitivity is the most important factor responsible for elevation of FPG levels in isolated IFG subjects.

## 1. Introduction

Impaired glucose tolerance (IGT) and impaired fasting glucose (IFG) are the different categories of abnormal glucose metabolism in the early stage development of type 2 diabetes. Two-hour PG elevation keeps steps with FPG elevation; however, the individuals showing dominant elevation of 2 h PG and showing dominant elevation of FPG exist. Elevation of 2 h PG and FPG levels are regulated by insulin secretory capacity and insulin sensitivity, but it is still controversial which factors are responsible for initial elevation of 2 h PG and FPG levels in the early stage development of type 2 diabetes. There were a few studies that directly compared pathophysiology between isolated IGT and isolated IFG to elucidate the differences of metabolic abnormality (isolated IGT is a subgroup of impaired glucose regulation showing only dominant elevation of 2 h PG, defined as FPG < 100 mg/dL within the normal range and 2 h PG 140–199 mg/dL within the range of borderline; isolated IFG is a subgroup of impaired glucose regulation showing only dominant elevation of FPG, defined as FPG 100–125 mg/dL within the range of borderline and 2 h PG < 140 mg/dL within the normal range), but it is not conclusive yet because the study conditions are different in subjects' ethnicity and methods examined. Weyer et al. reported isolated IFG and isolated IGT showed a similar impairment in insulin action but isolated IFG showed remarkable defect in early-phase insulin secretion in Pima Indian [1]. Festa et al. demonstrated that isolated IGT was more insulin resistant than isolated IFG in three ethnic populations: non-Hispanic whites, African Americans, and Hispanics [2]. We reported that impaired early-phase insulin secretion plays the more important role in deterioration from normal glucose tolerance (NGT) via isolated IGT to isolated postchallenge hyperglycemia (IPH) in Japanese [3]. In Korean subjects, pathogenesis of isolated IFG was associated with insulin resistance and isolated IGT was associated with impaired insulin secretion [4]. It is to be discussed whether decreased insulin secretory capacity and/or decreased insulin sensitivity play critical roles in the dominant elevation of 2 h PG and FPG levels in the early stage development of type 2 diabetes. Several studies have reported that there are some differences in atherogenic factors, such as triglyceride and apolipoprotein B and progression of atherosclerosis between subjects with IGT and IFG [5–10]. Previous epidemiological studies demonstrated that IGT has a higher risk for death from cardiovascular disease (CVD) compared with IFG [11–13]. Thus, it is also important to discuss the similarities and differences between isolated IGT and isolated IFG in view of future development of diabetic complications.

In the present study, we directly compared the clinical characteristics between isolated IGT and isolated IFG subjects and investigated the factors responsible for dominant elevation of 2 h PG and FPG levels in the prediabetic population. From the OGTT examinations, we divided subjects into subgroups by glucose tolerance, that is, isolated IGT and isolated IFG subjects, and compared the clinical parameters, indices of insulin secretory capacity, and insulin sensitivity. To determine the primary factors elevating 2 h PG or FPG levels in isolated IGT and isolated IFG groups, respectively, we analyzed the relationships between 2 h PG and clinical parameters in isolated IGT and between FPG levels and clinical parameters in isolated IFG subjects.

## 2. Subjects and Methods

We studied a total of 5620 cases who underwent 75 g oral glucose tolerance test (OGTT) owing to positive urine glucose test, >5.5% HbA1c level, >100 mg/dL fasting plasma glucose level, or family history of diabetes at initial examination for medical check-up from 1993 to 2013. From the beginning of 5620 individuals, 715 subjects were excluded because of FPG levels < 60 mg/dL, ≧126 mg/dL or 2 h PG levels < 60 mg/dL, ≧200 mg/dL for this study to analyze the factors in the early stage development of type 2 diabetes, having hypertension, hepatic, pancreatic, or renal dysfunction, endocrine or malignant disease, or history of heavy exercise, gastrectomy, or medication known to affect glucose metabolism, and 4905 subjects were included finally. This study was a cross-sectional, multicenter study at Kyoto University Hospital, Ikeda Hospital, Kansai Electric Power Hospital, Kansai Health Management Center, Center for Preventive Medicine of St. Luke's International Hospital, and Kyoto Preventive Medical Center.

We obtained fasting, 30, 60, and 120 min blood samples after oral administration of 75 g glucose for measurement of clinical parameter, plasma glucose, and serum insulin levels during 75 g OGTT. Standard OGTT with 75 g glucose was administered according to the National Diabetes Data Group recommendations [14], which require subjects to fast overnight for 10 to 16 hours before blood collection. We measured HbA1c, total-cholesterol, high-density lipoprotein cholesterol (HDL-cholesterol), and triglyceride (TG) levels.

Plasma glucose level was measured by glucose oxidase method using Hitachi Automatic Clinical Analyzer 7170 (Hitachi Co. Ltd., Tokyo, Japan). Serum insulin level was measured by chemiluminescent immunoassay (ARCHITECT insulin assay, Abbot Laboratories, Abbot Park, IL). Serum total-cholesterol and triglycerides levels were determined as reported previously [15]. HbA1c value, estimated as an NGSP equivalent value, was calculated by the following formula: HbA1c (JDS) + 0.4%. HbA1c was measured by HLC-723G7 (Tosoh, Tokyo, Japan) following the previous Japanese standard measurement methods [16]. Early-insulin secretion was calculated using the formula for insulinogenic index: [Insulin_30_ − Insulin_0_ (pmol/L)]/[Glucose_30_ − Glucose_0_ (mmol/L)] [17]. Whole-body insulin sensitivity was evaluated by ISI composite

(1) $$\begin{array}{c} \left(\;\text{composite index of insulin sensitivity}\;\right):\frac{10000}{\sqrt{\left({Glucose}_{0}\times {Insulin}_{0}\right)\times \left({\text{mean Glucose}}_{0-120}\times {\text{mean Insulin}}_{0\text{–}120}\right)}}. \end{array}$$

See [18]. In addition, estimate of insulin sensitivity was assessed by homeostasis model assessment-insulin resistance (HOMA-IR) using the following formula: Insulin_0_ (pmol/L) × Glucose_0_ (mmol/L)/22.5 [19]. Disposition index (DI) was expressed as the multiplex of the indices of insulin secretion and insulin sensitivity and calculated using the following formula: Insulinogenic index × ISI composite [20].

All subjects were divided into four subgroups according to the ADA criteria, with normal fasting glucose with normal glucose tolerance (NFG/NGT), FPG < 100 mg/dL, and 2 h PG < 140 mg/dL; isolated IFG, FPG 100–125 mg/dL, and 2 h PG < 140 mg/dL; isolated IGT, FPG < 100 mg/dL, and 2 h PG 140–199 mg/dL; IFG with IGT (IFG/IGT), FPG 100–125 mg/dL, and 2 h PG 140–199 mg/dL [21].

Simple linear regression analysis was conducted for all subjects to investigate the associations between 2 h PG in isolated IGT subjects or FPG levels in isolated IFG subjects and the other clinical factors such as age, BMI, plasma glucose level, serum insulin level, HbA1c, TG, total-cholesterol, HDL-cholesterol, insulinogenic index, and ISI composite in IFG/IGT subjects. ANOVA and Bonferroni post hoc test were used to compare differences between groups and *p* < 0.05 was considered as statistically significant. We carried out multivariate regression analysis to identify the independent predictors of 2 h PG in isolated IGT subjects or FPG levels in isolated IFG subjects and evaluated variables with *β*, and *p* < 0.05 was considered as statistically significant. All statistical analyses were performed using SPSS version 14.0 (SPSS, Chicago, IL). All data are shown in mean ± SD.

## 3. Results

### 3.1. Clinical Characteristics of Subjects

The subjects were 4905 in total (3038/1867; males/females); NGT, 2046 (1012/1034); isolated IFG, 1510 (1098/412); isolated IGT, 396 (210/186); IFG/IGT, 953(718/235), according to ADA criteria [21]. The mean age of the subjects was 54.8 ± 11.1 years, and BMI was 23.0 ± 3.2 kg/m^2^. Parameters for glucose metabolism as the mean FPG, 2 h PG levels, and HbA1c were 99.5 ± 10.0 mg/dL, 123.7 ± 29.7, and 5.95 ± 0.42, respectively.

### 3.2. Clinical Characteristics of Subject Groups with Isolated IFG or Isolated IGT

Comparison of the clinical and metabolic characteristics between isolated IFG (*n* = 1510) and isolated IGT (*n* = 396) groups is shown in Table 1. FPG, BMI, and fasting insulin were significantly higher in isolated IFG group than those in isolated IGT group (*p* < 0.0001). Two-hour PG, age, and HbA1c were significantly higher in isolated IGT group than those in isolated IFG group (*p* < 0.05). Two groups did not differ significantly in triglyceride, total-cholesterol, and HDL-cholesterol. Area under the curve of glucose (AUC-glucose) was significantly higher in isolated IGT subjects but area under the curve of insulin (AUC-insulin) of isolated IGT was significantly lower than that of isolated IFG (data not shown).  Figure 2 shows the comparison of insulin secretory capacity and insulin sensitivity. The insulinogenic index representing for early-phase insulin secretory capacity during 0–30 min after glucose load was significantly lower in isolated IGT group than that in isolated IFG group (0.43 ± 0.34 versus 0.50 ± 0.47, resp.; *p* < 0.01), when these groups were directly compared. The ISI composite representing for whole-body insulin sensitivity was significantly lower in isolated IFG group than that in isolated IGT group (6.87 ± 3.38 versus 7.98 ± 4.03, resp.; *p* < 0.0001). The HOMA-IR was significantly higher in isolated IFG group than that in isolated IGT group (1.58 ± 0.92 versus 1.19 ± 0.66, resp.; *p* < 0.0001). There was no significant difference in disposition index between isolated IGT and isolated IFG groups (2.94 ± 2.49 versus 2.92 ± 2.80, resp.; *p* = 0.68).

### 3.3. The Relationship of 2 h PG and FPG with Factors Responsible for Glucose Intolerance

**Figure 1:**
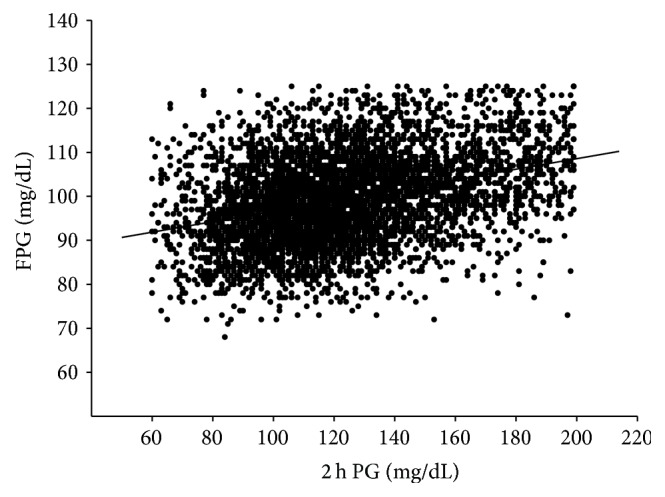
Simple linear regression line between 2 h PG and FPG levels based on least square method is indicated by solid line (*y* = 0.119*x* + 84.70). There was a positive correlation between 2 h PG and FPG significantly (*r* = 0.355, *p* < 0.0001).

**Figure 2:**
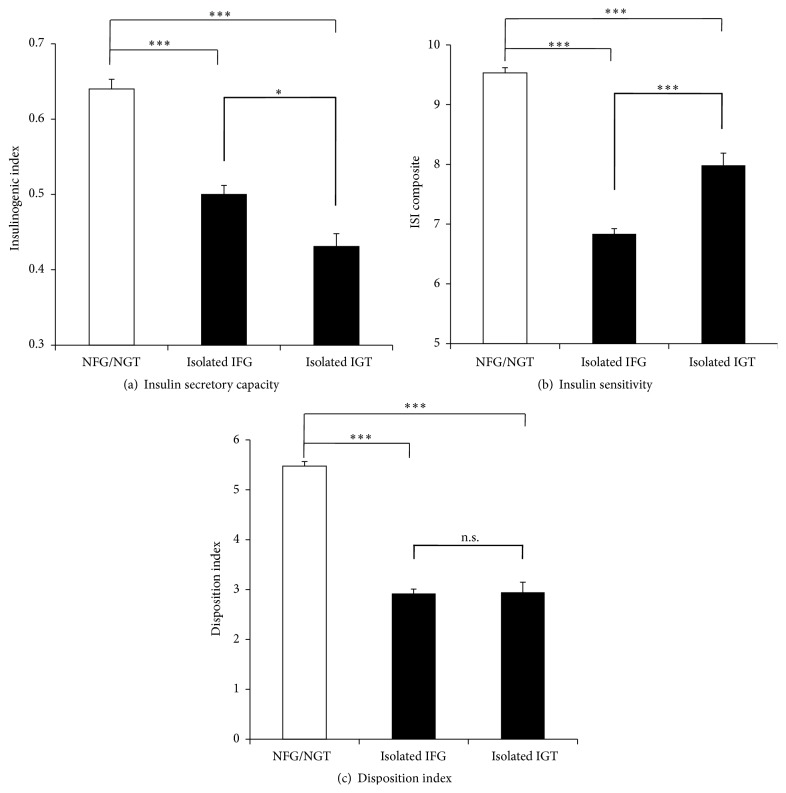
(a), (b), (c) Comparison of early-phase insulin secretory capacity (a), insulin sensitivity (b), and disposition index (c) among NFG/NGT, isolated IFG, and isolated IGT. Statistical significance between two groups was represented as ^*∗*^ : *p* < 0.05, ^*∗∗∗*^ : *p* < 0.0001, and n.s.: not significant.

The simple linear regression analysis between FPG (*y*-axis) and 2 h PG (*x*-axis) is shown in Figure 1. The resulting regression line was an equation of the first degree written as *y* = 0.119*x* + 84.70 (*r* = 0.355).  Table 2 shows the relationship of 2 h PG in IGT subjects and FPG in IFG subjects with factors responsible for glucose intolerance evaluated by simple linear regression analysis and multivariate regression analysis. In IGT subjects, insulinogenic index showed significant correlationship with 2 h PG levels in simple linear regression analysis and the highest *β*-value among independent variables associated with 2 h PG levels (*β* = −0.290, *p* < 0.0001). In IFG subjects, ISI composite, insulinogenic index, BMI, and TG showed significant correlationship with FPG levels in simple linear regression analysis and ISI composite showed the highest *β*-value among independent variables associated with FPG levels (*β* = −0.214, *p* < 0.0001) and insulinogenic index is the second only to ISI composite (*β* = −0.184, *p* < 0.0001).

## 4. Discussion

We examined the clinical characteristics of isolated IGT and isolated IFG in view of insulin secretory capacity and insulin sensitivity. In isolated IGT group, we have elucidated that early-phase insulin secretory capacity was the strongest factor to determine 2 h PG levels. Early-phase insulin secretory capacity of isolated IGT group was significantly lower than that of isolated IFG group. In isolated IFG group, insulin sensitivity was the strongest factor to determine FPG levels and early-phase insulin secretory capacity was also a strong factor to affect FPG levels next to insulin sensitivity. In isolated IFG group, insulin sensitivity was lower than that in isolated IGT group.

Different pathophysiology between isolated IGT and isolated IFG has been discussed but it is still under discussion. Controversial evidences in the previous studies may be because there are different characteristics of subjects including ethnicity and population of the subjects. In a Caucasian study, isolated IGT showing more decreased insulin secretion than isolated IFG, isolated IFG was more insulin resistant than isolated IGT [22]. In Pima Indian, non-Hispanic whites, African Americans, and Hispanics, isolated IGT was more insulin resistant than isolated IFG [1, 2]. But in Japanese and Korean, impaired early-phase insulin secretion plays the more important role in isolated IGT and insulin resistance plays more important role in isolated IFG subjects [3, 4, 23]. Another study reported that isolated IGT was characterized by both impaired insulin secretion and insulin resistance, but there are no significant differences in insulin sensitivity between isolated IFG and isolated IGT (*n* = 128, *n* = 55, resp.) subjects [24]. In the present study, we have elucidated the significant difference between isolated IGT and isolated IFG with large number of subjects. Early-phase insulin secretory capacity was lower in isolated IGT subjects than that in isolated IFG and insulin sensitivity was lower in isolated IFG subjects than that in isolated IGT subjects. Our data directly compared isolated IGT with isolated IFG and revealed for the first time the different characteristics of isolated IGT and isolated IFG in aspects of insulin secretory capacity and insulin sensitivity in Japanese.

Individuals with isolated IGT and isolated IFG both have higher risk for the development of type 2 diabetes than NFG/NGT [25]. However, large observational studies revealed that isolated IGT have higher CVD risk than isolated IFG [11–13]. In European study within NFG/NGT categories, elevated 2 h PG group (2 h PG levels were higher than FPG levels) showed higher mortality from CVD than the individuals whose 2 h PG returned to their FPG levels or lower [26]. In Japanese study within NFG/NGT, Morimoto et al. revealed that impaired insulin secretion group had a greater impact on the incidence of type 2 diabetes compared with insulin resistance group by large-scale prospective cohort study [27]. In the present study, early-phase insulin secretory capacity and AUC-insulin of isolated IGT were significantly lower than those of isolated IFG, and AUC-glucose of isolated IGT was significantly higher than that of isolated IFG. These results indicate that aggressive prevention of 2 h PG elevation is required from the aspects of complication and development of type 2 diabetes focusing on isolated IGT with stronger impairment of insulin secretion and glucose tolerance.

Two-hour PG levels showed the highest association with insulinogenic index in isolated IGT group in multivariate regression analyses. Fearch et al. concluded that a progressive and age-dependent loss of insulin secretion was involved in the development of postchallenge hyperglycemia in relation to progression from NGT to isolated IGT by 5-year follow-up study [28]. There are additional studies that reported that the main factors responsible for elevation of 2 h PG levels were decreased early-phase insulin secretion linked to aging [8, 29–31]. IGT subjects showed a larger decrease in insulin secretion compared with NGT subjects and early-phase was decreasing more than second-phase of insulin secretion [29]. When we compared isolated IGT and isolated IFG group, early-phase insulin secretion of isolated IGT group was lower than isolated IFG group, and mean age of isolated IGT group was higher than isolated IFG group. Together with these observations, age dependent *β*-cell dysfunction is possibly associated with decreased early-phase insulin secretion finally resulting in 2 h PG elevation.

FPG levels showed the highest association with ISI composite in isolated IFG group and secondly with insulinogenic index in multivariate regression analyses. Prospective study reported that insulin sensitivity in isolated IFG subjects was significantly reduced compared with that in NGT subjects prior to the development and diagnosis of isolated IFG [28, 32–34]. In this study, insulin sensitivity was lower in isolated IFG group than isolated IGT group and mean BMI of isolated IFG group was higher than isolated IGT group both within the normal range (BMI < 25). Thus, both decreased insulin sensitivity and decreased early-phase insulin secretory capacity play roles to elevate FPG levels but decreased insulin sensitivity in IFG subjects with higher BMI than isolated IGT has stronger effect on elevation of FPG levels than that of IGT subjects.

Decreased insulin secretory capacity had a stronger effect on 2 h PG elevation in the studies of Japanese, Korean, and Chinese subjects [3, 4, 35], while insulin resistance had a stronger involvement in 2 h PG elevation in other studies in Caucasian, Pima Indian, American, and Finnish studies [1, 2, 21, 36]. BMI of Japanese patients with type 2 diabetes shows only a little elevation (mean BMI was 23.1 kg/m^2^) from normal subjects (mean BMI: 22.7 kg/m^2^) in contrast with high BMI of Caucasian patients with type 2 diabetes (mean BMI: 29.4 kg/m^2^) [37]. Similar to Japanese, East-Asian patients with type 2 diabetes exhibited stronger association of insulin secretory capacity with glucose intolerance without morbid obesity [38]. There are ethnic differences in the contribution of insulin secretory capacity and insulin resistance to plasma glucose elevation and glucose intolerance as reported previously [3, 30, 39]. Further studies are required to establish whether similar results are observed in the other ethnic populations.

## 5. Conclusions

We have elucidated that isolated IGT has lower early-phase insulin secretory capacity than isolated IFG and isolated IFG has lower insulin sensitivity than isolated IGT.

Two-hour PG levels in isolated IGT group were strongly associated with decreased early-phase insulin secretory capacity. FPG levels in isolated IFG group were strongly associated with decreased insulin sensitivity and nextly with decreased early-phase insulin secretory capacity. Further studies are necessary to elucidate a strict causal relationship in a cohort study. The observation in the present study could be helpful for the prevention and treatment under the consideration of each individual's pathophysiology and phenotype in the early stage development of type 2 diabetes.

## Figures and Tables

**Table 1:** Clinical characteristics of subjects.

	NFG/NGT	Isolated IFG	Isolated IGT	IFG/IGT
Number (M/F)	2046 (1012/1034)	1510 (1098/412)	396 (210/186)	953 (718/235)
Age (yr)^*∗*^	53.0 ± 12.1	55.5 ± 10.0	57.1 ± 10.8	57.0 ± 9.9
BMI (kg/m^2^)^*∗∗*^	22.1 ± 3.0	23.5 ± 3.1	22.9 ± 3.3	24.3 ± 3.3
FPG (mg/dL)^*∗∗*^	91.0 ± 5.8	106.7 ± 5.6	93.2 ± 5.2	108.8 ± 6.4
2 h PG (mg/dL)^*∗*^	106.3 ± 18.0	112.8 ± 17.4	158.8 ± 15.2	164.0 ± 17.0
Fasting insulin (*μ*U/mL)^*∗*^	4.6 ± 2.2	6.0 ± 3.4	5.1 ± 2.8	7.0 ± 4.3
HbA1c (%)^*∗*^	5.80 ± 0.40	5.83 ± 0.43	5.93 ± 0.41	5.98 ± 0.44
Triglycerides (mg/dL)	91.4 ± 49.2	110.9 ± 61.0	105.5 ± 54.4	132.1 ± 73.9
Total-cholesterol (mg/dL)	208.7 ± 31.2	209.5 ± 31.2	209.5 ± 28.7	213.6 ± 31.5
HDL-cholesterol (mg/dL)	65.1 ± 16.1	61.9 ± 16.8	62.5 ± 16.0	57.6 ± 15.6

Clinical characteristics of subjects grouping by stage of glucose tolerance are listed. Data are mean ± SD. ^*∗*^ *p* < 0.05 and ^*∗∗*^ *p* < 0.0001 in ANOVA between isolated IGT and isolated IFG.

**Table 2:** Factors responsible for elevating FPG levels in isolated IFG and 2 h PG levels in isolated IGT.

Variable	Simple linear regression analysis		Multivariate regression analysis	
	Correlation coefficient	*p* value	*β*-value	*p* value
2 h PG levels as a dependent variable in isolated IGT				
Insulinogenic index	−0.245	*p* < 0.0001	−0.290	*p* < 0.0001
ISI composite	−0.015	ns	−0.097	ns
Age	0.043	ns	0.077	ns
Triglycerides	0.032	ns	0.053	ns
BMI	0.008	ns	0.007	ns

FPG levels as a dependent variable in isolated IFG				
ISI composite	−0.162	*p* < 0.0001	−0.214	*p* < 0.0001
Insulinogenic index	−0.111	*p* < 0.0001	−0.184	*p* < 0.0001
BMI	0.092	*p* < 0.0005	0.042	ns
Triglycerides	0.058	*p* < 0.05	0.038	ns
Age	0.028	ns	0.006	ns

Correlation coefficient and *p* values in simple linear regression analysis and *β*-values in multivariate regression analysis are listed. ns stands for not significant.
